# T-cell exhaustion in glioblastoma

**DOI:** 10.18632/oncotarget.26228

**Published:** 2018-10-19

**Authors:** Karolina Woroniecka, Peter E. Fecci

**Affiliations:** Preston Robert Tisch Brain Tumor Center, Duke University Medical Center, Durham, NC, USA; Department of Neurosurgery, Duke University Medical Center, Durham, NC, USA; Department of Pathology, Duke University Medical Center, Durham, NC, USA

**Keywords:** glioblastoma, immunotherapy, T-cell exhaustion, immune checkpoints, PD-1

Glioblastoma (GBM) is the most common primary malignant brain tumor and remains universally lethal. Median survival is persistently less than two years, despite modest improvements to standard of care. Immunotherapies, though FDA-approved in other solid tumors, are hampered in GBM by the tumor's marked heterogeneity and immunosuppressive influences. Propagating multiple modes of T-cell dysfunction facilitates GBM's ability to escape immunotherapeutic targeting [1, 2]. T-cell anergy [3] and tolerance [4, 5] are well-characterized in GBM patients, while other contributing manners of T-cell dysfunction, including sequestration [6], senescence, and exhaustion [7], are beginning to be further explored [8].

We uncovered recently that T-cell exhaustion likely makes substantial contributions to dysfunction among T-cells that successfully arrive at GBM, and may provide direct mechanistic limitations to the efficacy of checkpoint blockade [7]. Exhaustion is a hypo-responsive T-cell state resulting from chronic antigenic exposure under sub-optimal conditions. It was initially described amidst chronic viral infection (such as with chronic lymphocytic choriomeningitis virus (LCMV)) but is increasingly appreciated in cancer. Exhaustion represents a specific transcriptional program that is often characterized by up-regulation of the various co-inhibitory receptors constituting immune checkpoints. Blocking the classical immune checkpoints, PD-1 and CTLA-4, to rejuvenate T-cell function is an FDA-approved strategy in many cancers, yet a phase III clinical trial of PD-1 blockade demonstrated limited efficacy against GBM. Recent work has shown that one mode of resistance to PD-1 blockade involves the emergence of alternative immune checkpoints / exhaustion markers on T-cells, such as TIM-3 and LAG- 3 [9]. We therefore sought to determine the prevalence of exhaustion and these alternative immune checkpoints among T-cells in GBM.

We found that GBM does indeed elicit a severe exhaustion signature amidst T-cells [7]. T-cells infiltrating human GBM tumors (TIL) were found to express multiple immune checkpoints, including PD-1, TIM-3, LAG-3, TIGIT, and CD39. TIL were likewise less able to secrete the cytokines IFN-γ, IL-2, or TNF-α than T-cells isolated from patient or control blood. Among patient samples, triple-positive PD-1^+^TIM-3^+^LAG-3^+^ CD8^+^ T-cells were least functional compared to PD-1 negative and PD-1 single-positive cells, showing that mounting expression of the alternative immune checkpoints TIM-3 and LAG-3 resulted in loss of function. Importantly, we noted that CD8^+^ TIL expressing PD-1 alone remained functional, highlighting that expression of PD-1 alone may represent a state of activation rather than exhaustion.

In seeking to determine whether T-cell exhaustion arises preferentially in tumor-specific T-cells in human GBM, we found a surprising and disappointing lack of clonal expansion in human GBM-infiltrating CD8^+^ T-cells. Interestingly, CD4^+^ TIL demonstrated ample clonal expansion and, despite expressing multiple immune checkpoints, remained notably functional. These findings suggest a high level of CD8^+^ T-cell dysfunction that evolves immediately upon tumor infiltration (i.e. not after chronic exposure), imply differing susceptibilities of CD8^+^ and CD4^+^ T-cells to tumor-imposed dysfunction, and underscore the importance of functional studies over phenotype in proclaiming T-cell exhaustion.

We recapitulated our patient findings in two orthotopic murine models of GBM (SMA-560 and CT2A). Both models demonstrated high levels of triple-positive CD8^+^ TIL that were unable to secrete cytokines. Interestingly, the two tumors induced characteristically distinct exhaustion signatures, with CT2A upregulating PD1, TIM3, LAG3, TIGIT, CD39, and 2B4; while SMA- 560 consistently upregulated PD-1, TIM-3, LAG-3, CD39, and BTLA. Gene set enrichment analysis yielded that TIL were enriched for exhaustion genes and pathways that exactly matched patterns of LCMV viral-induced T-cell exhaustion. Therefore, T-cell exhaustive mechanisms may produce a varying cell surface phenotype, but exhaustion can and should be confirmed with functional and/or transcriptome (gene set) assays.

As we were able to track T-cells specific to the SMA-560 neoantigen ODC1 with a tetramer, we also examined the relative rate of exhaustion among tumor antigen-specific T-cells. ODC1 tetramer-positive CD8^+^ TIL were more likely to be PD1^+^TIM3^+^LAG3^+^ and less likely to be functional than ODC1 tetramer-negative CD8^+^ TIL demonstrating a propensity towards exhaustion in those T-cells possessing tumor specificity. This likewise implies that even if a faster process than seen in viral infection, tumor-induced exhaustion probably does require/follow antigenic exposure. What remains less clear is whether the source of such antigen is the tumor itself, antigen-presenting cells in the microenvironment, or some combination.

Interestingly, the consistent yet characteristically distinct exhaustion signatures elicited by CT2A and SMA-560 glioma models suggested that varying tumor types or locations might elicit different patterns of T-cell exhaustion. To evaluate, we implanted CT2A and SMA- 560 intracranial (ic) and subcutaneously (sc) and analyzed TIL at late stages of tumor growth. Surprisingly, the T-cell exhaustion signature remained constant for each glioma model, independent of the tumor's location. This finding was extended to tumors commonly metastatic to the brain, including melanoma (B16F10), breast carcinoma (E0771), and lung carcinoma (LLC). Each tumor yielded a characteristic and distinct TIL exhaustion signature that did not vary with ic or sc location. Importantly, CT2A and SMA-560 malignant gliomas induced the most severe exhaustion patterns and the poorest T-cell function of all tested tumor models.

Taken together, these findings suggest that exhaustion patterns among TIL (1) should be confirmed with functional or gene set analyses; (2) are determined by tumor-intrinsic factors and not environment; (3) strongly influence TIL function; and (4) vary in severity across and reflect tumor type, with gliomas inducing particularly severe T-cell exhaustion (Figure 1). Future studies will need to examine mechanisms of tumor-induced exhaustion, determine the limitations for checkpoint blockade, and assess the potential for T-cell rescue with rationally designed immunotherapeutic strategies.

**Figure 1:**
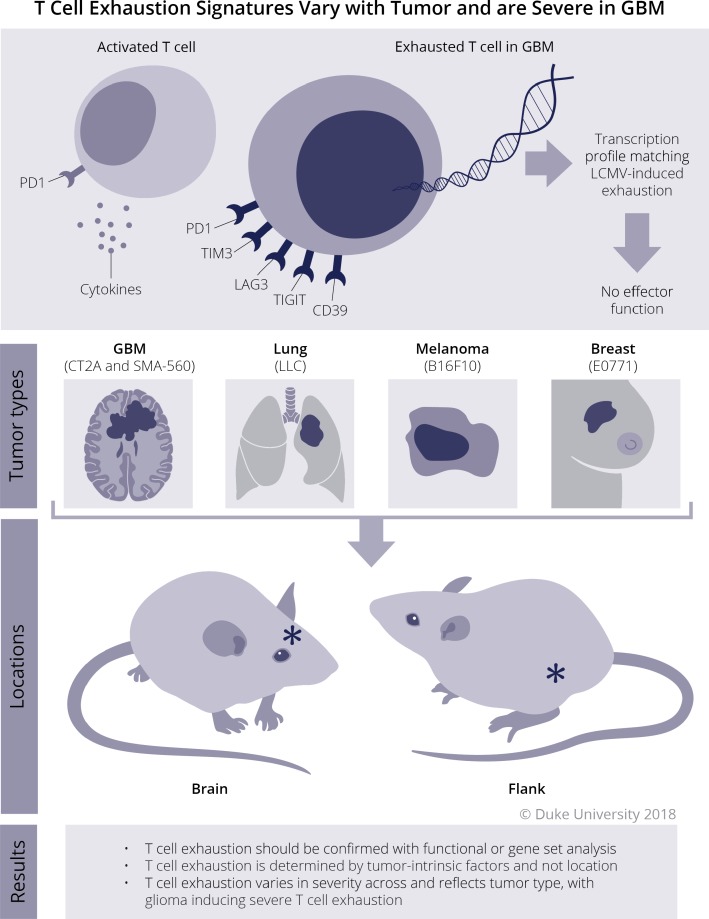
T cell exhaustion in glioblastoma Schematic representation of T-cell exhaustion dependence on tumor type, regardless of location.

## References

[1] Dix AR (1999). J Neuroimmunol.

[2] Fecci PE (2014). Clin Cancer Res.

[3] Elliott LH (1984). J Immunol.

[4] Fecci PE (2006). Cancer Res.

[5] El Andaloussi A (2006). Neuro Oncol.

[6] Chongsathidkiet P (2018). Nat Med.

[7] Woroniecka K (2018). Clin Cancer Res.

[8] Woroniecka KI (2018). Clin Cancer Res.

[9] Koyama S (2016). Nat Commun.

